# Comparative Performance of Pap Smear and Colposcopy in Cervical Cancer Screening Among Women With Abnormal Cervical Findings in Central India: A Prospective Observational Study

**DOI:** 10.1002/hsr2.72881

**Published:** 2026-07-30

**Authors:** Bhukya Priyanka, Bhakti Gurjar, Shreya Dahiwade, Gaurang Narayan, Calvin R. Wei, Babar Ali, Aymar Akilimali

**Affiliations:** ^1^ Department of Obstetrics and Gynaecology Indira Gandhi Government Medical College and Hospital Nagpur Maharashtra India; ^2^ Department of Obstetrics and Gynaecology Government Medical College & Hospital Nagpur Maharashtra India; ^3^ Department of Research and Development Shing Huei Group Taipei Taiwan; ^4^ Department of Research Medical Research Circle (MedReC) Goma DR Congo; ^5^ Faculty of Medicine University of Goma Goma DR Congo

**Keywords:** biopsy, cervical cancer, colposcopy, Pap smear, screening

## Abstract

**Background:**

Cervical cancer remains a leading cause of death among women worldwide, particularly in low‐ and middle‐income countries. This study aimed to evaluate and compare the effectiveness of Pap smear and colposcopy in detecting cervical abnormalities in women with unhealthy cervices and correlate findings with biopsy results.

**Methods:**

A prospective observational comparative study was conducted from July 2018 to December 2020. Women with abnormal cervical appearances or symptoms were enrolled. Each participant underwent a Pap smear followed by colposcopy, and cervical punch biopsies were performed on those with abnormal findings. Sensitivity, specificity, positive predictive value (PPV), negative predictive value (NPV), and diagnostic accuracy were calculated for both methods, using biopsy results as the gold standard.

**Results:**

Of the 82 participants, 74% were from rural areas, and the majority belonged to the lower‐middle socioeconomic class. Pap smear had a sensitivity of 77.14%, specificity of 63.64%, PPV of 87.1%, and NPV of 46.67%. Colposcopy showed a higher sensitivity of 90.91% but lower specificity of 37.5%, with a PPV of 85.71%. Diagnostic accuracy was 73.91% for Pap smear and 80.49% for colposcopy. Both methods were significantly associated with biopsy‐confirmed findings of cervical intraepithelial neoplasia (CIN) and invasive cancer.

**Conclusion:**

Pap smear and colposcopy are valuable screening tools, but their limitations underscore the need for supplemental diagnostic methods like biopsy, particularly in cases with abnormal findings. In low‐resource settings, innovative strategies such as “screen‐and‐treat” approaches should be considered to overcome barriers like follow‐up challenges and false‐negative rates, improving cervical cancer detection and prevention.

## 1 Introduction

Cervical cancer remains a leading cause of female mortality worldwide, with approximately 604,000 new cases and 342,000 deaths reported in 2020 [1]. The disease is particularly prevalent in low‐ and middle‐income countries, where over 90% of deaths occur [2]. In India, cervical cancer is the second most common cancer among women, contributing significantly to the global burden. In 2020, India reported 123,907 new cases and 77,348 deaths, with a 5‐year relative survival rate of about 46% [1, 3]. Cervical cancer, which develops from precancerous lesions caused by high‐risk HPV types, is preventable through early detection and treatment [4, 5]. Pap smears and HPV DNA testing are crucial for early diagnosis, but Pap smear alone has limitations, including low sensitivity and high false‐negative rates [5, 6]. Colposcopy, a noninvasive procedure that offers detailed visualization of the cervix, complements Pap smears by helping identify lesions and guiding biopsy [7].

While many studies have investigated the diagnostic efficacy of Pap smear and colposcopy techniques, their sensitivity and specificity have been highly variable depending upon study populations, disease prevalence, and healthcare settings. Most studies are conducted on well‐screened or controlled populations, which may not reflect real‐world conditions in low‐ and middle‐income countries. In India, a high prevalence of cervicitis, infections, and limited follow‐up may exert a strong effect on screening and diagnosis, particularly in resource‐limited settings where clinical care is constrained. Indeed, only very limited data are available on women with clinically unhealthy cervices, where diagnostic challenges are greater. Because of this reason, this study was carried out to reconsider and compare Pap smear and colposcopy's performance in the context of clinical practice in a real‐world tertiary care institution in Central India, and to verify their effectiveness with respect to histopathological findings in this target high‐risk group.

## 2 Methods

### 2.1 Study Structure and Participants

This prospective observational comparative study was carried out from July 2018 till December 2020 in the Gynaecology Outpatient Department of the tertiary care teaching hospital of Central India. Women aged ≥ 21 years and reporting regular cervical examination, or abnormal cervical characteristics or cervical complaints were systematically sampled. While the National Programme for Prevention and Control of Cancer, Diabetes, Cardiovascular Diseases and Stroke (NPCDCS) in India recommends population‐based screening for cervical cancer in women aged 30–65 years through visual inspection with acetic acid (VIA), the present study was a diagnostic accuracy study and not for population screening programs. Hence, all women who required clinical evaluation of an unhealthy cervix with age ≥ 21 years were included in the study to obtain a representation of normal gynecological practice during the time of the study. Pregnancy, prior treatment for cervical intraepithelial neoplasia (CIN) or cervical cancer, prior hysterectomy, active pelvic infection, and nonconsent were the exclusion criteria. Sample size estimation was generated by observing performance parameters related to diagnostic testing reported on by Nayani et al. [8]. A set sample size of around 80 people was deemed adequate to perform exploratory comparative analyses with 15% margin of error and 95% confidence level, taking into account expected sensitivity ranges of around 80%–90% for colposcopy and 30%–80% for Pap smear. In all, 82 women were included in the final sample.

### 2.2 Study Procedure

The ethics approval was obtained from the Institute Human Ethics Committee of Indira Gandhi Government Medical College Nagpur (IGGMC/Pharma/BORS/PB/OBGY/2018). All participants gave written informed consent, with additional permission before cervical punch biopsy if required. Confidentiality and anonymity of participants was preserved by data anonymization and password‐based data storage. We collected sociodemographic, clinical, and risk factor information using an appropriately validated structured proforma. All participants had Pap smear examination and colposcopy.

The cervical cytology was obtained with Ayre's spatula, fixed in alcohol and ether, and reported according to the Bethesda System. Colposcopy is used on the proMIS COLpro 222DX‐Ozview colposcope, at the time of commencing menstruation and usually daily from 10 to 12 days. After the addition of normal saline and 5% acetic acid, the cervical findings were recorded and assessed according to Reid's Colposcopic Index (RCI). Colposcopy‐directed punch biopsy was performed from abnormal acetowhite areas if any abnormalities were discovered on Pap smear, colposcopy, or clinical examination. The reference standard was histopathology.

We interpreted pap smear slides by a senior cytologist who was blinded to colposcopic and histopathological findings. For equivocal cases, secondary review by a senior cytopathologist was carried out, leading to a better diagnostic reliability. Colposcopic screenings were performed separately without information about cytology or biopsy findings. Because many slides were evaluated by a single primary observer and systematic dual review was not performed for all specimens, formal interobserver agreement analysis using kappa statistics could not be calculated. This is acknowledged as a limitation of the study.

### 2.3 Statistical Analysis of Data

Data were entered into Microsoft Excel and analyzed with SPSS version 22 (IBM Corp., Armonk, NY, USA). Colposcopic findings were categorized into normal, unsatisfactory, CIN I, CIN II/III, invasive cancer, or inflammatory/other lesions. Histopathological findings were categorized as normal, reparative changes, CIN I, CIN II, CIN III, microinvasive carcinoma, or invasive carcinoma. Frequencies and percentages were used to summarize categorical variables. Sensitivity, specificity, positive predictive value (PPV), negative predictive value (NPV), and diagnostic accuracy of Pap smear and colposcopy measurement were determined by histopathology (gold standard). We used McNemar's test to analyze paired diagnostic performance among 46 women who underwent biopsy, whereas when expected cell frequencies were small Fisher's Exact Test was used. Confidence intervals were computed at the rate of 95%, whereas a *p* < 0.05 was viewed as statistically significant.

## 3 Results

**Figure 1:**
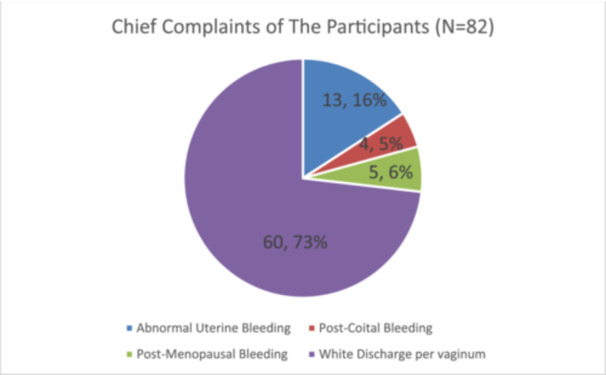
Distribution of presenting chief complaints of the study participants (*N* = 82).

The study included 82 participants with varying sociodemographic characteristics. Table 1 depicts the descriptive statistics regarding the sociodemographic characteristics of the study group. The majority of participants (61, 74.39%) resided in rural areas and belonged to the age group 31–40 years (32, 37.80%). Regarding socioeconomic status, the largest group (35, 42.28%) belonged to the lower‐middle class. Interestingly, Nullipara accounted only for 2.44% (*n* = 2) of the sample. A normal body mass index (BMI) was observed in 58.54% of the women, while 26.83% were overweight and 9.76% were classified as obese. Figure 1 demonstrates the most pressing chief complaint that made the study participants visit the gynecology clinic.

**Table 1:** Distribution of sociodemographic details of the study participants (*N* = 82).

S. No.	Characteristic	Subcategory	Frequency (%)	Mean ± SD	Median
1.	Age group	21–30 years	20 (25.60%)	39.72 ± 9	39 (39.5–45)
		31–40 years	32 (37.80%)		
		41–50 years	18 (21.95%)		
		51–60 years	12 (14.63%)		
2.	Residence	Rural	61 (74.39%)		
		Urban	21 (25.61%)		
3.	Education	Illiterate	31 (37.80%)		
		High school	38 (46.34%)		
		> 10th standard	13 (15.85%)		
4.	Socioeconomic status (Modified – Kuppuswamy's Scale)	Upper class	01 (01.22%)		
		Upper middle class	04 (04.88%)		
		Lower middle class	35 (42.28%)		
		Upper lower class	23 (28.05%)		
		Lower class	19 (23.17%)		
5.	Duration of marriage	< 10 years	17 (20.73%)	18.09 ± 9	16 (10.25–25.75)
		10–20 years	32 (39.02%)		
		> 20 years	33 (40.24%)		
6.	Distribution of parity	Nullipara	02 (02.44%)		
		P1	08 (09.76%)		
		P2	38 (46.34%)		
		P3	26 (31.71%)		
		P4	06 (07.32%)		
		P5	02 (02.44%)		
		> P5	00		
7.	Menstrual phase	Reproductive age	52 (63.41%)		
		Perimenopausal	17 (20.73%)		
		Postmenopausal	13 (15.85%)		
8.	Body mass index (BMI) (in Kg/m^2^) (Quetlet's Index)	Underweight (< 18.5)	04 (04.88%)	23.56 ± 3.73	23 (21–25)
		Normal (18.50–22.99)	48 (58.54%)		
		Overweight (23.00–24.99)	22 (26.83%)		
		Obese Category I (25.00–29.99)	08 (09.76%)		
		Obese Category II (> 30.00)	00		

Table 2 depicts the distribution of findings from per‐speculum examination, Pap smear test, colposcopy findings, and cervical punch biopsy. Pap smear results revealed that 39.02% (*n* = 32) of participants had inflammatory findings. Squamous cell carcinoma was identified in 2.44% (*n* = 2) of the participants. Colposcopy findings showed 20.73% (*n* = 17) exhibiting signs consistent with CIN 1, and 21.95% (*n* = 18) with CIN 2/3. Cervical punch biopsies were performed on 46 participants who tested positive in either Pap smear, colposcopy, or both. Cervicitis was diagnosed in 21.74% (*n* = 10) of biopsied patients, while CIN 1 and CIN 2/3 were found in 45.65% (*n* = 21) and 26.09% (*n* = 12) of cases, respectively. A paired comparison analysis using McNemar's test among the 46 biopsy‐confirmed cases demonstrated that colposcopy identified significantly more true positive lesions than Pap smear (McNemar *p* < 0.05), confirming that the diagnostic sensitivity advantage of colposcopy over Pap smear was statistically significant.

**Table 2:** Distribution of findings from per‐speculum examination, Pap smear test, colposcopy findings, and cervical punch biopsy.

S. No.	Characteristic	Characteristic finding	Frequency (%)
1.	Per‐speculum exam	Normal finding	10 (12.19%)
		White discharge	09 (10.97%)
		Polyp	02 (02.43%)
		Congestion	12 (14.63%)
		Erosion	29 (35.36%)
		Nabothian cysts	04 (04.87%)
		Hypertrophy	13 (18.85%)
		Candidiasis	01 (01.21%)
		Mucopurulent discharge	02 (02.43%)
		Total	82 (100%)
2.	Pap smear test	Negative for intraepithelial lesion (NIEL)	06 (07.31%)
		Inflammatory (INF)	32 (39.02%)
		Inflammatory squamous metaplasia (INF‐SM)	07 (08.53%)
		Inflammatory *Candida* Species (INF‐CS)	03 (03.65%)
		Inflammatory bacterial vaginosis (INF‐BV)	02 (02.43%)
		Inflammatory trichomoniasis vaginosis (INF‐TV)	01 (01.22%)
		Low‐grade squamous intraepithelial lesion (LSIL)	15 (18.29%)
		High‐grade squamous intraepithelial lesion (HSIL)	11 (13.41%)
		Atypical squamous cells of undetermined significance (ASCUS)	03 (03.66%)
		Squamous cell carcinoma (SCC)	02 (02.44%)
		Total	82 (100%)
3.	Colposcopy	Normal	04 (04.88%)
		Inflammation/erosion	38 (46.34%)
		Hazy/faint acetowhite area, fine punctations or mosaicism, Iodine partial positivity (CIN 1)	17 (20.73%)
		Dense acetowhite areas, coarse punctations or mosaicism, iodine‐negative epithelium (CIN 2/3)	18 (21.95%)
		Unsatisfactory	05 (06.10%)
		Total	82 (100%)
4.	Cervical punch biopsy^a^	Normal	01 (02.17%)
		Cervicitis	10 (21.74%)
		Microinvasive carcinoma (MIC)	02 (04.35%)
		Cervical intraepithelial neoplasia (CIN 1)	21 (45.65%)
		Cervical intraepithelial neoplasia CIN 2/3	12 (26.09%)
		Total	46 (100%)

^a^ Only 46 patients of the 82 (56.09%) who screened positive either in Pap smear/colposcopy/both were subjected to cervical punch biopsy.

Table 3 shows that Pap smear and colposcopy findings were significantly associated with cervical punch biopsy results, with a notable detection of CIN 1 and CIN 2/3 in 45.65% and 21.09% of cases, respectively. Fisher's Exact Test indicated a highly significant association between screening modalities and biopsy findings (*p* < 0.0001).

**Table 3:** Association of Pap smear result and colposcopy with punch biopsy findings (*N* = 46).

S. No.	Pap smear/colposcopy (*N*)	Normal	Cervicitis	CIN 1	CIN 2/3	MIC	Total	*p*
1.	Pap smear results							
	Negative for intraepithelial lesion (NIEL)	00	00	01	00	00	01	< 0.0001*
	Inflammatory (INF)	01	06	06	01	00	14	
	Low‐grade squamous intraepithelial lesion (LSIL)	00	03	11	01	00	15	
	High‐grade squamous intraepithelial lesion (HSIL)	00	00	01	10	00	11	
	Atypical squamous cells of undetermined significance (ASCUS)	00	01	02	00	00	03	
	Squamous cell carcinoma (SCC)	00	00	00	00	02	02	
	Total	00	10	00	00	02	46	
2.	Colposcopy							
	Inflammation/erosion	00	03	03	00	00	06	< 0.0001*
	Hazy/faint acetowhite area, fine punctations or mosaicism, iodine partial positivity (CIN 1)	00	05	11	01	00	17	
	Dense acetowhite areas, coarse punctations or mosaicism, iodine‐negative epithelium (CIN 2/3)	00	00	05	11	02	18	
	Unsatisfactory	01	02	02	00	00	05	
	Total	01	10	21	12	02	46	

* Fischer's Exact Test applied.

Table 4 summarizes the sensitivity, specificity, PPV, and NPV of pap smear result and colposcopy considering biopsy as gold standard. Diagnostic accuracy was assessed by comparing pap smear and colposcopy results with biopsy findings. Pap smear had a sensitivity of 77.14% and a specificity of 63.64%, while colposcopy demonstrated a higher sensitivity of 90.91%, but a lower specificity of 37.50%. Colposcopy achieved a diagnostic accuracy of 80.49%, outperforming Pap smear at 73.91%.

**Table 4:** Sensitivity, specificity, positive predictive value, and negative predictive value of Pap smear result and colposcopy considering biopsy as gold standard (taking 95% confidence interval).

Variables	Sensitivity (95% CI)	Specificity (95% CI)	AUC	PPV (95% CI)	NPV (95% CI)	Diagnostic accuracy (95% CI)
Pap smear	77.14% (62.7–87.9)	63.64% (30.8–89.1)	0.70	87.10% (73.6–94.5)	46.67% (23.6–70.0)	73.91% (59.8–85.1)
Colposcopy	90.91% (78.3–96.7)	37.50% (13.7–69.4)	0.64	85.71% (72.5–93.3)	50% (26.0–74.0)	80.49% (67.1–89.6)

Abbreviations: AUC, area under curve; PPV, positive predictive value; NPV, negative predictive value.

## 4 Discussion

Cervical cancer remains a major public health challenge in developing countries such as India, where cytology‐based screening programs have had limited success despite the potential to reduce disease incidence by up to 80% through effective screening, coverage, and follow‐up. Limited trained personnel, inadequate laboratory facilities, high costs, and poor follow‐up continue to hinder conventional screening strategies, highlighting the need for effective diagnostic approaches in low‐resource settings [2, 4, 7].

Most participants were aged 31–40 years (mean age 39.7 years), presented with white discharge per vaginum (73%, *n* = 60), resided in rural areas (74%, *n* = 61), and belonged to the lower‐middle socioeconomic class (42%, *n* = 35). A substantial proportion were illiterate (37.80%, *n* = 31) or educated below the 10th grade (46.34%, *n* = 38). These findings are consistent with previous studies by Malur and colleagues, Agrawal and colleagues, Prasad and colleagues, and Kushtangi and colleagues, emphasizing the influence of sociodemographic factors on cervical cancer screening and prevention [9, 10, 11, 12].

Pap smear demonstrated moderate diagnostic performance, with a sensitivity of 77.14%, specificity of 63.64%, PPV of 87.1%, NPV of 46.67%, and diagnostic accuracy of 73.91%. These findings are comparable to those reported by Malur et al., Nkwabong et al., and Kharkwal et al. [9, 13, 14]. The relatively lower specificity observed in our study may be attributable to the high prevalence of infection and inflammatory cervical changes, methodological differences, and the limited sample size, which may have contributed to false‐positive results and reinforced the need for histopathological confirmation in women with abnormal cervical findings.

Colposcopy demonstrated higher sensitivity (90.91%) but lower specificity (37.5%) than Pap smear, with a PPV of 85.71% and diagnostic accuracy of 80.49%. These findings are comparable to those reported by Olaniyan, Ali, Tawa et al., and Singh et al. [15, 16, 17, 18]. Colposcopy rarely achieves 100% sensitivity or specificity because its performance depends on operator interpretation, lesion characteristics, and underlying cervical pathology. Inflammatory cervicitis, benign squamous metaplasia, and reactive epithelial changes may produce acetowhite areas that mimic CIN, resulting in false‐positive impressions and reduced specificity. Importantly, colposcopy should not be regarded as a confirmatory diagnostic test; rather, it serves as a diagnostic evaluation and biopsy‐guidance tool. Definitive diagnosis requires histopathological confirmation, which explains the use of biopsy as the reference standard in this study. Therefore, while colposcopy offers superior sensitivity for lesion detection, it is best utilized as a complementary triage modality alongside cytology rather than as a standalone replacement for Pap smear.

### 4.1 Strengths

The study's strengths lie in its prospective and comparative design, which enhances the reliability of the findings by providing a comprehensive evaluation of Pap smear and colposcopy for cervical cancer detection. The use of cervical punch biopsy as the gold standard adds robustness to the analysis, allowing for a clear assessment of diagnostic accuracy. Conducted in a real‐world, low‐resource setting in Central India, the study reflects conditions faced by underserved populations, making its findings highly relevant and applicable. Comprehensive data collection, including sociodemographic factors, clinical history, and risk profiles, offers a deeper understanding of the relationship between socioeconomic conditions and screening outcomes. By focusing on women with unhealthy cervices, such as those with chronic cervicitis, lesions, and polyps, the study mirrors the complexity of real clinical scenarios, providing practical data for improving screening strategies in resource‐constrained environments. The novelty of the study lies in its focus on low‐resource settings, comparative analysis of screening methods, and integration of colposcopy and Pap smear to improve diagnostic accuracy. Additionally, the study highlights the diagnostic challenges posed by high infection rates in such settings and offers insights into the influence of sociodemographic factors on screening outcomes, contributing valuable knowledge to cervical cancer prevention efforts in low‐ and middle‐income countries.

### 4.2 Limitations

The limitations of this study, including the lack of HPV testing and incomplete cervical biopsies, must be acknowledged. Biopsies were only performed in cases with abnormal Pap smear or colposcopy results or in patients with persistent symptoms, leading to a reduced sample size for biopsy‐confirmed diagnoses. This limitation may have contributed to the lower specificity of the Pap smear and the moderate NPV observed. Additionally, the high prevalence of infections and inflammations in this population may have further increased the rate of false‐negative results. In resource‐limited settings like India, where follow‐up can be challenging due to financial, cultural, or logistical barriers, the need for alternative screening methods that are both accurate and feasible becomes evident.

In low‐resource countries, where regular hospital attendance and follow‐up are often inconsistent, alternative strategies such as the “screen‐and‐treat” approach have been shown to be effective. This method, which bypasses the need for extensive follow‐up by treating patients immediately after screening, may offer a practical solution for improving cervical cancer prevention in such settings. The adoption of more reliable screening methods, combined with immediate treatment, when necessary, could significantly reduce the burden of cervical cancer in these populations.

## 5 Conclusion

This study highlights the importance of Pap smear and colposcopy as essential tools for cervical cancer screening in low‐resource settings. Although both methods have demonstrated reasonable sensitivity and specificity, limitations such as false negatives, over‐reporting, and sample size constraints indicate the necessity for supplementary diagnostic tools like biopsy, particularly in cases with abnormal findings. The study's results emphasize the challenges posed by sociodemographic factors, infection prevalence, and follow‐up limitations in resource‐poor regions. To improve early detection and treatment of cervical cancer, innovative approaches, including “screen‐and‐treat” strategies, are crucial for more accurate and timely interventions in such settings. Colposcopy offers greater sensitivity but also requires histopathological confirmation to avoid false positives. Colposcopy demonstrated higher sensitivity than Pap smear in detecting CIN; however, given its lower specificity, it should be utilized as a complementary adjunct and triage tool rather than a replacement for Pap smear screening. Integrating both modalities together can optimize diagnostic yield and reduce missed high‐grade disease, particularly in low‐resource settings where maximizing screening efficacy is essential. Ultimately, the success of cervical cancer prevention programs in low‐resource settings will depend on the development and implementation of screening methods that are both accurate and accessible, alongside improved follow‐up and treatment protocols. In the Indian public health system context, strengthening Pap smear‐based primary screening with selective colposcopy triage for abnormal results may optimize resource utilization, reduce unnecessary interventions, and provide a more feasible screening strategy for cervical cancer prevention in resource‐limited settings. Future research should explore integrated Pap smear + colposcopy triage algorithms with selective incorporation of HPV testing to strengthen cervical cancer screening pathways in India.

## Author Contributions

**Bhukya Priyanka:** conceptualization, methodology, supervision, visualization, resources, writing – review and editing, writing – original draft, formal analysis, software, investigation, data curation. **Bhakti Gurjar:** conceptualization, software, methodology, data curation, investigation, supervision, formal analysis, resources, writing – original draft, writing – review and editing, visualization. **Shreya Dahiwade:** data curation, software, formal analysis, writing – original draft, investigation, visualization, resources. **Gaurang Narayan:** software, methodology, investigation, formal analysis, project administration, resources, writing – review and editing, supervision, writing – original draft. **Calvin R. Wei:** writing – review and editing, visualization, project administration, resources, software, validation. **Babar Ali:** validation, writing – review and editing, project administration, formal analysis, investigation. **Aymar Akilimali:** methodology, supervision, investigation, validation, formal analysis, writing – review and editing, resources, project administration, visualization, software. All authors have read and approved the final version of the manuscript.

## Funding

The authors have nothing to report.

## Ethics Statement

Institute Human Ethics Committee approval (IEC/BORS) of Indira Gandhi Government Medical College, Nagpur bearing Reference Number IGGMC/Pharma/BORS/PB/OBGY/2018 was duly obtained prior to start of the study. The study design incorporated the separation of patient identification data by confidentiality codes to maintain anonymity and protect the privacy of the participants. This study was performed in accordance with the ethical standards as laid in the 1964 Declaration of Helsinki and its later amendments or comparable ethical standards.

## Consent

Informed consent was obtained from all participants involved in the study. Written informed consent was obtained from the patient for the research and the publication in the local vernacular language, Hindi/Marathi.

## Conflicts of Interest

The authors declare no conflicts of interest.

## Transparency Statement

Bhukya Priyanka affirms that this manuscript is an honest, accurate, and transparent account of the study being reported; that no important aspects of the study have been omitted; and that any discrepancies from the study as planned have been explained.

## Data Availability

Master sheet of the collected data and other supplementary material including clinical proforma used, consent forms are available. They can be provided if requested for. We affirm that this manuscript is an honest, accurate, and transparent account of the study being reported; that no important aspects of the study have been omitted; and that any discrepancies from the study have been explained. Bhukya Priyanka and Gaurang Narayan have full access to all of the data in this study and take complete responsibility for the integrity of the data and the accuracy of the data analysis.

## References

[1] H. Sung , J. Ferlay , R. L. Siegel , et al., “Global Cancer Statistics 2020: GLOBOCAN Estimates of Incidence and Mortality Worldwide for 36 Cancers in 185 Countries,” CA: A Cancer Journal for Clinicians 71 (2021): 209–249, 10.3322/caac.21660. 33538338

[2] Global Cancer Observatory , *Estimated Cancer Incidence, Mortality and Prevalence Worldwide in 2018: Cervical Cancer* (International Agency for Research on Cancer, World Health Organization, 2018), https://gco.iarc.fr/today/data/factsheets/cancers/23-Cervix-uteri-fact-sheet.pdf.

[3] F. Bray , J. Ferlay , I. Soerjomataram , R. L. Siegel , L. A. Torre , and A. Jemal , “Global Cancer Statistics 2018: GLOBOCAN Estimates of Incidence and Mortality Worldwide for 36 Cancers in 185 Countries,” CA: A Cancer Journal for Clinicians 68, no. 6 (2018): 394–424, 10.3322/caac.21492. 30207593

[4] J. S. Berek , Berek and Novak's Gynecology, 13th ed. (Lippincon Williams and Wilkins, 2002), 11.99.

[5] S. Wardak , “Human Papillomavirus (HPV) and Cervical Cancer,” Medycyna Doświadczalna i 68: 73. 28146625

[6] M. M. Patel , A. N. Pandya , and J. Modi , “Cervical Pap Smear Study and Its Utility in Cancer Screening, to Specify the Strategy for Cervical Cancer Control,” National Journal of Community Medicine 2 (2011): 49–51.

[7] S. K. Das , S. Nigam , A. Batra , and M. Chandra , An Atlas of Colposcopy, Cytology and Histopathology of Lower Female Genital Tract (CBS Publishers and Distributors, 2008), 2–3.

[8] Z. S. Nayani and P. C. Hendre , “Comparision and Correlation of Pap Smear With Colposcopy and Histopathiology in Evaluation of Cervix,” Journal of Evolution of Medical and Dental Sciences 4, no. 53 (July 2015): 9236–9247, 10.14260/jemds/2015/1341.

[9] P. Malur , B. R. Desai , A. Dalal , et al., “Sequential Screening With Cytology and Colposcopy in Detection of Cervical Neoplasia,” Journal of SAFOG 1: 45–48, 10.5005/jp-journals-10006-1009.

[10] A. Agrawal , A. Sharma , M. Gupta , and N. Agarwal , “Role of Cytology, Colposcopy and Colposcopic Directed Biopsy in the Evaluation of Unhealthy Cervix,” International Journal of Reproduction, Contraception, Obstetrics and Gynecology 5 (2016): 3765–3769.

[11] D. Prasad , A. Sinha , U. Mishra , S. Parween , R. B. Raman , and N. Goel , “Colposcopic Evaluation of Cervix in Symptomatic Women and Its Correlation With Pap Smear. A Prospective Study at a Tertiary Care Centre,” Journal of Family Medicine and Primary Care 10, no. 8 (August 2021): 2923–2927, 10.4103/jfmpc.jfmpc_1208_20. PMC8483131 34660425

[12] P. Kushtagi and P. Fernandez., “Significance of Persistent Inflammatory, Cervical Smears in Sexually Active Women of Reproductive Age,” Journal of Obstetrics and Gynecology of India 52, no. 1 (January–February 2002): 124–126.

[13] E. Nkwabong , I. Laure Bessi Badjan , and Z. Sando , “Pap Smear Accuracy for the Diagnosis of Cervical Precancerous Lesions,” Tropical Doctor 49, no. 1 (January 2019): 34–39, 10.1177/0049475518798532. 30222058

[14] K. C. Kharkwal , K. Lo , A. Tahmina , et al., “A Study on Effectiveness of Pap Smear in Mass Screening of Premalignant Lesions of Cervix,” SBV Journal of Basic, Clinical and Applied Health Science 2, no. 2 (2019): 65–68.

[15] O. B. Olaniyan , “Validity of Colposcopy in the Diagnosis of Early Cervical Neoplasia—A Review,” African Journal of Reproductive Health 6, no. 3 (December 2002): 59–69. 12685410

[16] B. Ali , “Evaluation of the Frequency and Patterns of Cervical Cancer Recurrence After Treatment Using ^18F‐FDG PET‐CT: A Cross‐Sectional Study,” Health Science Reports 8, no. 11 (2025): e71566, 10.1002/hsr2.71566. 41292574 PMC12641100

[17] K. Tawa , A. Forsythe , J. K. Cove , A. Saltz , H. W. Peters , and W. G. Watring , “A Comparison of the Papanicolaou Smear and the Cervigram: Sensitivity, Specificity, and Cost Analysis,” Obstetrics and Gynecology 71, no. 2 (February 1988): 229–235. 3336558

[18] S. L. Singh , N. A. Dastur , and M. S. Nanavatti , “A Comparison of Colposcopy and Pap Smear: Sensitivity, Specificity and Predictive Values,” Bombay Hospital Journal 42, no. 4 (2000).

